# Solvothermal Synthesis, Structure and Optical Property of Nanosized CoSb_3_ Skutterudite

**DOI:** 10.1007/s11671-010-9700-4

**Published:** 2010-07-28

**Authors:** Latha Kumari, Wenzhi Li, JianYu Huang, Paula P Provencio

**Affiliations:** 1 Department of Physics, Florida International University, Miami, FL 33199 USA; 2 Sandia National Laboratories, Center for Integrated Nanotechnologies (CINT), Albuquerque, NM 87185 USA

**Keywords:** Nanostructures, Chemical synthesis, Electron microscopy, Optical properties

## Abstract

Binary skutterudite CoSb_3_ nanoparticles were synthesized by solvothermal method. The nanostructuring of CoSb_3_ material was achieved by the inclusion of various kinds of additives. X-ray diffraction examination indicated the formation of the cubic phase of CoSb_3_. Structural analysis by transmission electron microscopy analysis further confirmed the formation of crystalline CoSb_3_ nanoparticles with high purity. With the assistance of additives, CoSb_3_ nanoparticles with size as small as 10 nm were obtained. The effect of the nanostructure of CoSb_3_ on the UV–visible absorption and luminescence was studied. The nanosized CoSb_3_ skutterudite may find application in developing thermoelectric devices with better efficiency.

## Introduction

Novel thermoelectric (TE) materials are potential candidates for power generation and solid-state cooling applications as they can directly convert thermal energy into electrical energy or vice versa [1]. A TE device has no moving parts, produces no noise, has high reliability, and exhausts no waste. The performance of TE device can be quantified by the dimensionless figure of merit *ZT* = (*α* ^2^ *σ*/*κ*)*T*, where *α* is the Seebeck coefficient, *σ* and *κ* are the electrical and thermal conductivities, respectively, and *T* is the temperature in Kelvin. Among a number of TE materials investigated, the family of skutterudites is regarded as a class of promising TE materials with high performance because these compounds are typical phonon glass and electron crystal (PGEC) materials [2, 3]. Skutterudites have excellent thermoelectric properties at high temperature and offer the opportunity of building thermoelectric devices operational at room temperature. The binary skutterudites can be represented by a formula, MX_3_ (M=Co, Rh, Ir; X=P, As, Sb), and they have a cubic structure and a space group *Im* 3 symmetry. Of the different types of binary skutterudites, CoSb_3_ has attracted the greatest interest, because it not only exhibits some of the best thermoelectric properties but also has abundant supply for its constituent elements that are less volatile and less expensive elements than those used for other skutterudite compounds [4]. CoSb_3_ is a narrow band-gap semiconductor, and its transport properties have been studied [5, 6].

CoSb_3_ is promising for thermoelectric applications due to its high Seebeck coefficient and high electrical conductivity which give rise to a good ZT of about 1 [2, 6–8]. However, its high thermal conductivity makes it difficult to be an efficient thermoelectric material [9]. In attempts to lower the thermal conductivity, techniques such as nanostructuring [4, 9], rare earth metal filling [10, 11], doping [12], and nanoparticle dispersion of CoSb_3_[13, 14] have been developed. These modifications [10–14] to the CoSb_3_ matrix are expected to potentially reduce the thermal conductivity of the composites via the enhancement of the phonon scattering. One of the remarkable features of CoSb_3_ skutterudite is the cage-like open structure, which can be filled with foreign atoms acting as phonon rattlers [15]. The “rattling” of the filled atoms scatters phonons strongly and drastically reduces the thermal conductivity of the skutterudite compounds [10, 16, 17]. As a result, the decrease in thermal conductivity can improve the efficiency of the thermoelectric device. Various kinds of rare earth elements such as Ba, Ce, La, Ca, [8, 18–20], and Yb [7, 21, 22] have been used to fill the cages, thereby resulting in an improved ZT. Yb is one of the ideal filler or rattler species, and it has been widely studied [7, 21, 22]. Nolas et al*.* reported Yb-filled *n*-type Yb_0.19_Co_4_Sb_12_ with a peak ZT close to 1 at 373°C [7], Geng et al. presented Yb_0.15_Co_4_Sb_12_ with ZT of about 0.7 at 400°C [21], and Yang et al. [23] achieved a ZT of about 1.2 at 550°C in Yb_0.35_Co_4_Sb_12_.

Another effective approach for achieving a lower thermal conductivity of CoSb_3_ skutterudite is through nanostructuring, which means reducing the grain size of the TE material down to nanoscale. Nanostructured materials have attracted much focus compared to their bulk counterparts due to their fascinating physical, optical, electrical, and thermoelectric properties as well as their potential applications in nanodevices [24]. If a bulk material is composed of nanoparticles, the decrease in grain size for the nanoparticles leads to a drastic increase in the density of grain boundaries, which can result in a typical density of 10^19^ interfaces per cubic centimeter. The increased grain boundaries in nanocrystalline materials cause large changes in the physical properties compared with that in micrometer-sized polycrystals [25]. Recently, theoretical predictions have shown that the nanostructuring of TE materials produces higher grain-boundary and shorten phonon mean free path, which results in a significant reduction in thermal conductivity due to the stronger selective scattering of phonons than that of charge carriers [26–29]. The nanosized CoSb_3_ materials also show potential as a possible anode material for Li-ion batteries [30].

CoSb_3_ skutterudite materials are generally processed by synthesis techniques such as mechanical alloying [31], ball milling [31], arc melting [32], chemical alloying [33], solid-state reaction [34], ultrasonic spray pyrolysis [35], co-precipitation [33], sol–gel [36], and solvothermal method [37, 38]. Especially, the solvothermal method is a simple and effective way for the synthesis of nanostructured materials and has advantages such as its relatively low processing temperature, high reproducibility, low cost, large-scale production, and its ability to control the size and shape of the material with the assistance of suitable additives. A high reaction temperature of 240°C, long reaction duration of 72 h, and multiple reaction steps are essential in the solvothermal synthesis [37, 38]. In the present work, the solvothermal synthesis of CoSb_3_ nanomaterials is presented. Surface morphology and crystal structure variation of the synthesized materials with the addition of different surfactants and polymer have been discussed. In particular, the effect of addition of surfactant, sodium dodecyl sulfate (SDS), on the surface morphology and crystal structure as well as the optical properties of the CoSb_3_ is discussed in detail.

## Experiments

### Synthesis of CoSb_3_ Nanoparticles

Analytically pure CoCl_2_·6H_2_O and SbCl_3_ (Fisher Scientific) in a molar ratio of 1:3 were used as the starting materials without further purification. The starting materials were placed in Teflon-lined autoclave that was later filled with ethanol up to 80% of its total volume. A sufficient amount of NaBH_4_ as reducing agent was added into the Teflon-liner, and the reduction reaction lasted for 15–20 min. Then, the autoclave was sealed and maintained at 240°C for 72 h. Once the reaction finished, the autoclave was cooled to room temperature naturally. The reaction precipitate was then filtered, washed several times with distilled water and ethanol, and dried at 100°C for 4 h. The above synthesis procedure was repeated with the addition of various surfactants used as structure directing/capping agents: 0.25 mmol of sodium dodecyl sulfate (SDS), 0.25 mmol of Cetyl trimethylammonium bromide (CTAB), and 1 ml of Triton X-100. The CoSb_3_ samples were also prepared with 0.25 mmol of Poly(vinyl pyrrolidone) (PVP) as a mild reducing agent and stabilizer. The CoSb_3_ samples produced with SDS, CTAB, Triton, and PVP are termed as CoSb_3_-SDS, CoSb_3_-CTAB, CoSb_3_-Triton, and CoSb_3_-PVP, respectively. The CoSb_3_ sample synthesized without using any additive is named as CoSb_3_-NON.

### Characterization

X-ray diffraction measurements were taken using Siemens D5000 diffractometer equipped with Cu anode operated at 40 kV and 40 mA. The XRD patterns were collected with a step size of 0.01° and a scan rate of 1 s/step. Surface morphology analysis of the CoSb_3_ materials was performed by a field emission scanning electron microscope (SEM, JEOL JSM-6330F, 15 kV). Transmission electron microscopy (TEM) and high-resolution TEM (HRTEM) images, selected-area electron diffraction (SAED) patterns, and energy-dispersive X-ray spectroscopy (EDS) spectrum were obtained from a FEI Tecnai F30 apparatus operated at an accelerating voltage of 300 kV with a point-to-point resolution of 2 Å. UV–visible spectra were obtained from a Perkin-Elmer Lambda 900 UV/Vis/NIR spectrometer, and the photoluminescence spectra were recorded from a Horiba Jobin–Yvon FluoroLog FL3-22 spectrofluorometer. For the spectroscopic analysis, CoSb_3_ materials were dispersed in NaOH solution at room temperature and the solution was taken into a quartz cell (1 cm optical path length).

## Results and Discussion

### X-ray Diffraction

**Figure 1:**
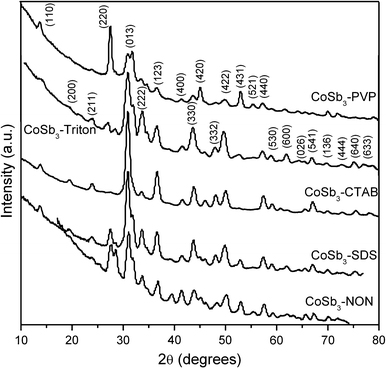
XRD profiles of the CoSb_3_ nanoparticles synthesized with or without additives. All the diffraction peaks can be indexed to cubic phase of CoSb_3_

Figure 1 shows the XRD patterns of the cobalt antimonide materials synthesized by the solvothermal route with and without additives. The diffraction peaks in all the XRD spectra can be indexed as binary skutterudite CoSb_3_ with cubic phase, space group *Im* 3, and lattice constant of *a* = 0.904 nm. The XRD spectra match very well with the standard XRD file (JCPDS File: 65-3144) of the cubic CoSb_3_. The synthesis reactions for the formation of CoSb_3_ can be written as:
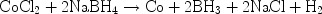





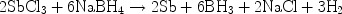





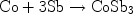



The above reaction mechanism indicates the stepwise formation of the CoSb_3_ phase. In the beginning of the reaction process, the strong reducing agent NaBH_4_ rapidly and completely reduces the Co^2+^ and Sb^3+^ ions to Co and Sb atoms as indicated by reaction Eq. 1 and 2, respectively. Earlier reports [37, 39] on the synthesis of CoSb_3_ nanoparticles indicated the formation of CoSb and CoSb_2_ as intermediate phases before the formation of final CoSb_3_ phase. In the present work, no clear peaks for possible intermediate phases of CoSb_2_ or Sb were noticed in the XRD pattern (Fig. 1), indicating the formation of pure phase of CoSb_3_ (reaction (3)) [40]. A previous report by Mi et al. [37] suggests that a synthesis temperature of around 250°C with long reaction duration is necessary for obtaining pure phase of CoSb_3_ without the impurities Sb and CoSb_2_, and the impurities will be formed at low processing temperature and short duration. Hence, in the present work, the absence of the intermediate products (Sb and CoSb_2_) [40] can be attributed to the reaction temperature of 240°C and the prolonged synthesis duration of 72 h. The current XRD result and the reported works [37–40] reveal that the synthesis temperature and duration are key parameters in determining the phase composition of the samples.

Compared to the XRD spectrum of CoSb_3_ particles prepared without additive, the XRD spectra of the CoSb_3_ nanoparticles synthesized with various additives show peak broadening and small shift of diffraction peaks toward lower angles, which can be ascribed to the lattice orientation or rearrangement [41]. The peak shift and peak broadening can also be attributed to the internal strain in the crystal structure due to the stacking faults, grain boundaries, and small crystallites [41]. Surfactants typically play crucial roles in controlling the particle size and size distribution. The addition of surfactant as capping agent and structure directing agent in the synthesis process results in monodispersed and small-size nanoparticles [42]. In addition, the surfactants used for the nanoparticle synthesis can also induce oxide or amorphous layer surrounding the nanoparticles, which is expected in the materials synthesized by hydrothermal or solvothermal route [43]. The oxide or amorphous layer covering the outer surface of the nanoparticles can effectively influence the crystal structure of the nanoparticles as reflected by the peak shift and peak broadening in their XRD spectra [42]. More information on the crystal structure and oxide layer formation can be obtained from the TEM analysis.

### Surface Morphology

**Figure 2:**
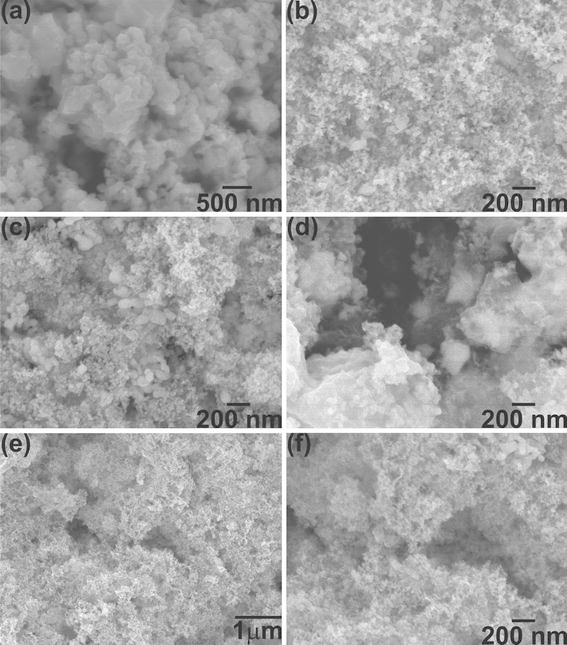
SEM images of the CoSb_3_ nanoparticles synthesized with or without additives. **a** CoSb_3_-NON, synthesized without additive. **b** CoSb_3_-SDS, synthesized with SDS. **c** CoSb_3_-CTAB, synthesized with CTAB. **d** CoSb_3_-Triton, synthesized with Triton. **e** and **f** CoSb_3_-PVP, synthesized with PVP

Figure 2 presents the SEM images of the CoSb_3_ nanoparticles synthesized with and without additives as structure directing and capping agents. The CoSb_3_-NON sample synthesized without using additive (Fig. 2a) has a particles size of around 50-100 nm where most of the granules form clusters and have irregular shapes [37, 38]. Figure 2b and c shows the SEM images of samples synthesized with SDS and CTAB, respectively. The samples synthesized with surfactants show a reduced particle size in the range of 10 nm. Sample synthesized with CTAB (Fig. 2c) shows the presence of some big particles with the size in the range of 50–100 nm, but the density of the big particles is low. The CoSb_3_ material synthesized with Triton shows large clusters of nanoparticles with irregular shape (Fig. 2d). Figure 2e and f represent the low- and high-magnification images of the CoSb_3_ sample, respectively, prepared with PVP as both structure directing and mild reducing agent. The CoSb_3_ sample synthesized with PVP as additive consists of nanoparticles with the size of around 10 nm and has a uniform size distribution. It was reported that high-purity nanocrystalline CoSb_3_ nanoparticles with the size below 30 nm were synthesized via a modified polyol process with PVP and tetra-ethylene glycol as stabilizer and solvent, respectively, at 240°C for 15 min [39]. However, there is no report on the synthesis of CoSb_3_ nanoparticles with the addition of surfactants as presented in this work. From the SEM results, it can be concluded that the use of additives induces the reduction in the particle size. However, due to the nanosize of the particles, it is not hard to explain the exact shape and size of the nanoparticles from the SEM images. Further, TEM analysis is performed on the nanoparticles to obtain more information about the structure of the particles.

### TEM Analysis

**Figure 3:**
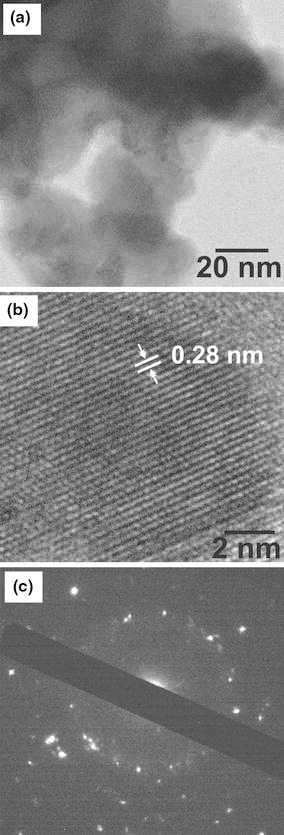
TEM examination results of the CoSb_3_-NON sample. **a** TEM image, **b** high-resolution TEM image, and **c** SAED pattern

**Figure 4:**
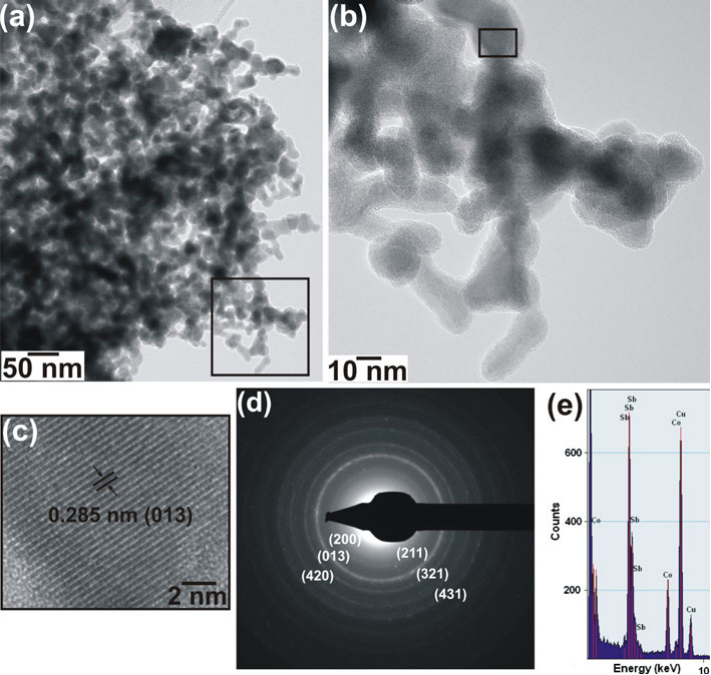
TEM examination results of the CoSb_3_-SDS nanoparticles. **a** Low-magnification image. **b** High-resolution TEM image of the boxed area in **a**. **c** A close-up of the boxed area in (**b**), showing lattice fringes of the nanocrystal. **d** SAED pattern. **e** EDS spectrum

TEM analysis of the CoSb_3_ material prepared without using additive (CoSb_3_-NON) is performed. The high-magnification TEM image of CoSb_3_ sample (Fig. 3a) shows nanoparticles with a size of 50–100 nm. HRTEM image of a single nanoparticle in Fig. 3b shows equally spaced and clear lattice fringes separated by a distance of 0.28 nm that corresponds to the interplanar distance of (013) plane of the cubic CoSb_3_. SAED image (Fig. 3c) of CoSb_3_-NON sample indicating clear ring patterns can be assigned to the various lattice planes of the cubic CoSb_3_. Figure 4 presents the TEM images of the CoSb_3_-SDS nanoparticles synthesized by the solvothermal method with additive SDS. The low-magnification TEM image of CoSb_3_-SDS sample in Fig. 4a shows that the as-prepared sample is composed of peanut-like nanoparticles with width of 10–12 nm and length of about 25–40 nm. The nanoparticles are of irregular shape and connected to each other to form peanut-like structure as shown in the high-magnification TEM image (Fig. 4b), which corresponds to the region marked by an open box in Fig. 4a. The nanoparticles synthesized in the present work are much smaller in size when compared to the particles prepared by the solvothermal method in the reported works [37–39]. The enlarged view of the image indicated by open box in Fig. 4b is presented as a HRTEM image in Fig. 4c showing the lattice fringes with separation distance of 0.285 nm that can be attributed to the interplanar distance of (013) plane of cubic CoSb_3_. Figure 4d shows the SAED image with the clear ring patterns, which can be indexed to the various lattice planes of the cubic CoSb_3_. The possible chemical composition of the as-synthesized nanoparticles can be obtained from EDS spectrum as shown in Fig. 4e. The EDS indicates that the as-prepared material is made up of Co and Sb. The Cu peak in the EDS exists due to the supporting copper TEM grid.

Further, the TEM analysis of CoSb_3_-SDS nanoparticles (Fig. 4) reveals the influence of the addition of SDS on the synthesis of the nanoparticles. The closer view of the CoSb_3_-SDS nanoparticles in Fig. 4b indicates the formation of thin layer of about few nm over the peanut-like nanoparticles, and the thin layer can be assigned to the oxide or amorphous layer. The effect of SDS inclusion on the crystal structure of CoSb_3_-SDS nanoparticles can also be understood by comparing the SAED patterns of CoSb_3_-NON and CoSb_3_-SDS samples in Figs. 3c and 4d, respectively. The CoSb_3_-NON sample (Fig. 3c) shows spot pattern, while the CoSb_3_-SDS (Fig. 4d) presents discrete ring pattern. The occurrence of the ring pattern of the CoSb_3_-SDS nanoparticles can be attributed to the SDS addition and induced formation of oxide layer surrounding the nanoparticles. XRD analysis also confirms the influence of the SDS on the crystal structure of the CoSb_3_-SDS sample.

### Optical Characterization

The optical properties, for example, the strong nonlinear optical response, of semiconductor nanomaterials have attracted the attention of many researchers because of their potential applications [44, 45]. Research results show that the electronic and optical properties of the semiconducting nanoparticles are influenced by both their size and shape [46–48]. The ability to tune their absorption and photoluminescence spectra over a wide range of energy by varying the crystal size provides the opportunity of fabricating nanocrystal-based tunable lasers and light-emitting diodes. It has been predicted that the binary skutterudite, CoSb_3_, is a narrow band-gap semiconductor [47] and its energy gap falls in the far-infrared region. However, in the present work, the optical characterization is limited in the UV–visible region.

**Figure 5:**
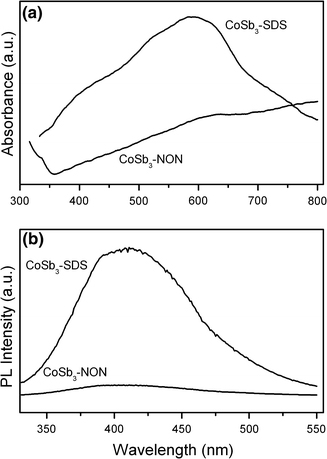
**a** UV–visible absorption spectra and **b** PL spectra of the CoSb_3_-NON and CoSb_3_-SDS samples

The optical properties of the CoSb_3_ nanoparticles synthesized with and without additive are analyzed by UV–visible absorption and photoluminescence spectra. Figure 5a presents the UV–visible absorption spectra recorded in the wavelength region of 300–800 nm for CoSb_3_-NON and CoSb_3_-SDS samples at room temperature. The optical absorption spectra of CoSb_3_-NON and CoSb_3_-SDS samples show enhanced absorption in the low wavelength region, which can be attributed to the absorption in the band-gap region. The CoSb_3_-NON sample indicates the absorption peak maximum at about 640 nm. The CoSb_3_-SDS sample presents a broad-band absorption spectrum with the peak maximum around 600 nm, which also indicates the blue-shift with respect to CoSb_3_-NON sample. Sometimes the broadening of the absorption spectrum and peak shift can occur due to the quantum confinement of the nanoparticles [49]. The position of the absorption maximum is affected by the decrease in particle size. However, both a blue-shift and a red-shift have been observed with decreasing particle size [50]. The addition of surfactants in the nanoparticles synthesis can also induce defects or defaults on the surface of the nanoparticles, which affects the optical properties. The enhanced absorption in the low-energy region (visible region) for CoSb_3_-SDS nanoparticles (Fig. 5a) can be assigned to the trap states or defect states in the band-gap region that arise due to the effect of surfactants on the surface of the nanoparticles. Sofo et al. [47] reported the ab initio calculations which showed that the CoSb_3_ is a typical narrow band-gap semiconductor. The gap is strongly dependent on the relative position of the Sb atoms inside the unit cell. A band gap of 0.22 eV was obtained after minimizing these positions. This value is more than four times larger than the result of a previous calculation, which reported that the energy bands near the Fermi surface are unusual [51]. The temperature dependence of the far-infrared reflection spectra of CoAs_3_ showed an enhanced free carrier contribution, and an energy gap of 0.21 eV was estimated from the optical measurement [52].

The photoluminescence spectra were obtained in the wavelength region of 330–550 nm for the CoSb_3_-NON and CoSb_3_-SDS samples at room temperature. From the PL spectra in Fig. 5b, it is evident that the CoSb_3_-SDS sample shows broad emission spectrum with enhanced intensity when compared to that of the CoSb_3_-NON sample. The emission maximum for the CoSb_3_-NON and CoSb_3_-SDS samples are at 411 and 409 nm, respectively. The slight blue-shift of emission band observed for the CoSb_3_-SDS sample can be attributed to the decrease in the particle size [49, 50]. The broad band at the interface of the UV and visible region can be assigned to both inter-band transition and defect-related transition. The origin of defects can be ascribed to the solvothermal synthesis of CoSb_3_, where the nanostructured intermediate products provide the defects as the high-diffusivity paths for the formation of CoSb_3_[37]. The addition of surfactants can also induce the formation of defect or trap states in the band-gap region, which can give rise to the enhanced emission in the low-energy region. XRD and TEM analyses also confirm the surfactant-induced formation of oxide or amorphous structure on the outer surface of the nanoparticles. Photoluminescence from self-assembly of Ge nanoclusters grown on Si(100) via a buffer layer-assisted growth method [53] was expected to arise from localized luminescence centers that originate from defect centers at the Ge/Si interface or defect centers inside the Ge clusters. The strong and sharp PL bands were observed in the near infrared spectral region for samples with different cluster sizes. To the best of the authors’ knowledge, optical characterizations of CoSb_3_ skutterudites are seldom reported. Hence, a detailed investigation into the fundamental optical mechanism in CoSb_3_ is essential.

## Conclusions

The skutterudite CoSb_3_ nanoparticles were synthesized by solvothermal route with or without using additives. The structural analysis confirms the formation of pure cubic phase of CoSb_3_. Uniform CoSb_3_ nanoparticles with width of about 10 nm are obtained with the addition of additives. A broad photoluminescence band with maximum intensity at 409 nm was observed for CoSb_3_ nanoparticles synthesized with sodium dodecyl sulfate. Comparing with the CoSb_3_ nanoparticles synthesized without additive, the CoSb_3_ nanoparticles synthesized with the sodium dodecyl sulfate show enhanced photoluminescence. The nanosized skutterudite CoSb_3_ synthesized by solvothermal method could be used to develop high-efficiency thermoelectric devices.

## References

[1] DiSalvo FJ (1999). Science.

[2] Sales BC, Mandrus D, Chakoumakos BC, Keppens V, Thompson JR (1997). Phys. Rev. B.

[3] Nolas GS, Morelli DT, Tritt TM (1999). Annu. Rev. Mater.Sci.

[4] Toprak M, Stiewe C, Platzek D, Williams S, Bertini L, Müller E, Gatti C, Zhang Y, Rowe M, Muhammed M (2004). Adv. Funct. Mater.

[5] Uher C, Tritt TM (2001). Skutterudites: prospective nobel thermoelectric semiconductors and
semimetals.

[6] Caillat T, Borshchevsky A, Fleurial J-P (1996). J. Appl. Phys.

[7] Nolas GS, Kaeser M, Littleton RT, Tritt TM (2000). Appl.Phys. Lett.

[8] Chen LD, Kawahara T, Tang XF, Goto T, Hirai T, Dyck JS, Chen W, Uher C (2001). J. Appl. Phys.

[9] Mi JL, Zhu TJ, Zhao XB, Ma J (2007). J. Appl. Phys.

[10] Sales BC, Mandrus D, Williams RK (1996). Science.

[11] Liu K, Dong X, Jiuxing Z (2006). Mater. Chem. Phys.

[12] Chitroub M, Besse F, Scherrer H (2009). J. Alloys Compd.

[13] Li JQ, Feng XW, Sun WA, Ao WQ, Liu FS, Du Y (2008). Mater. Chem. Phys.

[14] He Z, Stiewe C, Platzek D, Karpinski G, Müller E, Li S, Toprak M, Muhammed M (2007). Nanotechnology.

[15] Slack GA, Rowe DM (1995). CRC Handbook of Thermoelectrics.

[16] Nolas GS, Cohn JL, Slack GA (1998). Phys. Rev. B.

[17] Tritt TM (2001). Semiconductors and Semimetals.

[18] Chen B, Xu JH, Uher C, Morelli DT, Meisner GP, Fleurial JP, Caillat T, Borshchevsky A (1997). Phys. Rev. B.

[19] Keppens V, Mandrus D, Sales BC, Chakoumakos BC, Dai P, Coldea R, Maple MB, Gajewski DA, Freeman EJ, Bennington S (1998). Nature (London).

[20] Puyet M, Lenoir B, Dauscher A, Dehmas M, Stiewe C, Muller E (2004). J. Appl. Phys.

[21] Geng HY, Ochi S, Guo JQ (2007). Appl. Phys. Lett.

[22] Li H, Tang XF, Zhang QJ, Uher C (2008). Appl. Phys. Lett.

[23] Yang J, Hao Q, Wang H, Lan YC, He QY, Minnich A, Wang DZ, Harriman JA, Varki VM, Dresselhaus MS, Chen G, Ren ZF (2009). Phys. Rev. B.

[24] Xia Y, Yang P, Sun Y, Wu Y, Mayers B, Gates B, Yin Y, Kim F, Yan H (2003). Adv. Mater.

[25] Siegel RW, Fougere GE, Hadjipanayis GC, R.W. Siegel (1994). Mechanical Properties of Nanophase materials, in Nanophase
Materials.

[26] Gleiter H (2000). Acta Mater.

[27] Rowe DM, Bhandari CM (1980). Appl. Energy.

[28] Mahan GD, Ehrenreich H, F. Speapen (1997). in Solid State Physics.

[29] Goldsmid HJ, Sharp J, H.J. Goldsmid, G. Nolas (2000). in Proceedings of 18th International Conference on
Thermoelectrics.

[30] Xie J, Zhao XB, Cao GS, Zhao MJ, Su SF (2005). J. Power Sources.

[31] Yang J, Chen Y, Peng J, Song X, Zhu W, Su J, Chen R (2004). J. Alloys Compd.

[32] Kawaharada Y, Kurosaki K, Uno M, Yamanaka S (2001). J. Alloys Compd.

[33] Wang M, Zhang Y, Muhammed M (1999). Nanostruct. Mater.

[34] Chapon L, Ravot D, Tedenac JC (1999). J. Alloys Compd.

[35] Wojciechowski KT, Morgiel J (2003). in Proceedings of 22nd International Conference on
Thermoelectrics.

[36] Chu Y, Tang X, Zhao W, Zhang Q (2008). Cryst. Growth Des.

[37] Mi JL, Zhao XB, Zhu TJ, Tu JP, Cao GS (2006). J. Alloys Compd.

[38] Xie J, Zhao XB, Mi JL, Cao GS, Tu JP (2004). J. Zhejiang Univ. SCI.

[39] Yang L, Hng HH, Cheng H, Sun T, Ma J (2008). Mater. Lett.

[40] Li S, He Z, Toprak M, Stiewe C, Müller E, Muhammed M (2007). Phys. Stat. Sol. (RRL).

[41] Ungár T (2004). Scr. Mater.

[42] Teng X, Yang H (2004). J. Mater. Chem.

[43] Xie Y, Lu J, Yan P, Jiang X, Qian Y (2000). J. Solid State Chem.

[44] Alivisatos AP (1996). Science.

[45] Schmitt-Rink S, Miller DAB, Chemla DS (1987). Phys. Rev. B.

[46] Efros AL, Rosen M (1998). Phys. Rev. B.

[47] Sofo JO, Mahan GD (1998). Phys. Rev. B.

[48] Henglein A (1993). J. Phys. Chem.

[49] Koch U, Fojtik A, Weller H, Henglein A (1985). Chem. Phys. Lett.

[50] Kreibig U, Genzel U (1985). Surf. Sci.

[51] Singh DJ, Pickett WE (1994). Phys. Rev. B.

[52] Kliche G, Bauhofer W (1988). J. Phys. Chem. Solids.

[53] Li AP, Flack F, Lagally MG, Chisholm MF, Yoo K, Zhang Z, Weitering HH, Wendelken JF, Chisholm MF (2004). Phys. Rev. B.

